# TLR4 as a therapeutic target: Antidepressant mechanism of saikosaponin A in regulating the NF-κB/BDNF axis and mitigating oxidative stress and inflammation *in vivo* and *in vitro*

**DOI:** 10.3389/fphar.2025.1585290

**Published:** 2025-05-16

**Authors:** Lin Tan, Jiayue Li, Dingcheng Sun, Xinyi Tian, Xiaoli Zhong, Yiyi Shan

**Affiliations:** ^1^ Department of Urology Surgery and Department of Nursing, People’s Hospital of Deyang City, Deyang, Sichuan, China; ^2^ College of Food Science, Northeast Agricultural University, Haerbin, China; ^3^ Guangdong Pharmaceutical University, Guangzhou, China; ^4^ College of Chemistry and Pharmacy, Northwest A&F University, Xianyang, China; ^5^ Yixing Eye Hospital, Yixing, China

**Keywords:** depression, saikosaponin A, TLR4/NF-κB/BDNF axis, neuroinflammation, oxidative stress

## Abstract

Natural plant-derived active ingredients have gained increasing attention for their potential in the treatment of depression due to their safety and multi-target pharmacological activities. Saikosaponin A (SSA), a major bioactive saponin extracted from *Bupleurum* (a medicine and food homologous plant), has been reported to possess anti-inflammatory, neuroprotective, and antioxidative properties. In this study, we evaluated the antidepressant-like effects of SSA in a mouse model of chronic unpredictable mild stress (CUMS)-induced depression. Mice were subjected to CUMS, followed by daily administration of SSA (20 mg/kg, po for 6 weeks). Behavioral tests, including tail suspension test, open field test, elevated plus maze, and marble burying test, indicated that SSA significantly alleviated depressive-like and anxiety-like behaviors. Histopathological analysis by H&E staining showed that SSA reduced hippocampal neuronal damage induced by chronic stress. Biochemical assays revealed that SSA normalized levels of neurotransmitters (5-HT, DA, and 5-HIAA), enhanced antioxidant enzyme activity (SOD, CAT, and GSH), and suppressed neuroinflammatory cytokine production (TNF-α, IL-1β, and IL-6). Mechanistically, SSA exerted its antidepressant effects by inhibiting the TLR4/NF-κB signaling pathway and upregulating BDNF expression. In PC12 cells, TLR4 overexpression attenuated SSA’s protective effects, whereas TLR4 silencing enhanced cellular resistance to corticosterone-induced damage. These findings suggest SSA alleviates CUMS-induced depression-like behaviors in mice by modulating neuroinflammation and oxidative stress through the TLR4/NF-κB/BDNF signaling axis, indicating its potential as a functional food-derived therapy for depression.

## Introduction

Depression is a neurological disease characterized by emotional loss, which endangers the physical and mental health of patients (Simon et al., 2024). According to the World Health Organization (WHO), by 2030, depression will become a major global disease burden and a major cause of disability (Wang et al., 2019). Despite considerable research, the pathophysiological mechanisms underlying depression remain complex and not fully understood. Growing evidence suggests that inflammation and oxidative stress are pivotal factors in the onset and progression of depression, and the two mechanisms often promote each other, exacerbating the condition (Neto et al., 2024). The occurrence of depression and the lack of therapeutic effect of antidepressants may be related to inflammation and oxidative stress. *Polygonatum sibiricum* polysaccharide mitigates depression-caused behavioral disorders and neuronal damage through its antioxidant and anti-inflammatory functions (Shen et al., 2021). Ginsenoside-Rg1 inhibits neuronal deterioration caused by stress-induced depression by preventing oxidative stress and inflammatory responses (Li et al., 2020). *Saccharum japonicum* ethanol extract exerts antidepressant/anxiety effects by mediating inflammation, oxidative stress, and apoptosis (Hao et al., 2021). Therefore, targeting inflammation and oxidative stress presents a promising therapeutic strategy for depression.

Bupleurum, a well-known traditional Chinese medicine, has long been used to treat various psychiatric disorders, including depression (Yang L. et al., 2020). Saikosaponin A (SSA) is a saponin substance found in Bupleurum that exerts antidepressant, anti-inflammatory, antioxidant, and brain-protective pharmacological activities (Sun et al., 2024). For instance, SSA has been shown to alleviate depression-like behavior in cerebral ischemia models by modulating the p-CREB/BDNF axis and inhibiting apoptosis (Wang et al., 2021). SSA recused perimenopausal depression-caused behavioral deficits by inhibiting the activation of the HPA axis, relieving neuroinflammation, and increasing BDNF expression (Chen et al., 2018). Although these studies highlight SSA’s antidepressant potential, its precise molecular targets and underlying mechanisms remain unclear.

Toll-like receptor 4 (TLR4), a member of the Toll-like receptor family, plays a key role in mediating inflammatory responses by promoting the production of various pro-inflammatory mediators (Zhang et al., 2022). TLR4 signaling pathway plays a crucial role in the development of several neuropsychiatric disorders (Saleki et al., 2024), such as schizophrenia (Jameie et al., 2025) and depression (Wei et al., 2025). Recent research has increasingly focused on TLR4 as a key therapeutic target due to its regulation of neuroinflammation and oxidative stress. For example, Suanzaoren decoction exerts antidepressant effects through the gut–brain axis, which inhibits TLR4/NF-κB/NLRP3-mediated neuroinflammation and improves colon pathological morphology and intestinal flora dysbiosis (Du et al., 2023). Oxysophoridine exerts the efficacy of alleviating Alzheimer’s disease and can inhibit Aβ-induced BV-2 cell injury, oxidative stress, and inflammation through the TLR4/NF-κB pathway (Chen et al., 2021). Arctigenin alleviated *Toxoplasma gondii*-caused depressive-like behavior by regulating TLR4/NF-κB axis-mediated microglial activation and neuroinflammation in BV2 cells and brain tissue (Cheng et al., 2020). These findings underscore the pivotal role of TLR4 signaling in depression and suggest that targeting this pathway could offer a novel therapeutic strategy. However, previous studies did not investigate the involvement of the TLR4 signaling pathway, and whether SSA can regulate the TLR4/NF-κB/BDNF axis has not yet been systematically explored. To our knowledge, this is the first study to comprehensively examine the regulatory effects of SSA on the TLR4/NF-κB/BDNF axis in both *in vivo* and *in vitro* models of depression. By combining behavioral assessments and molecular investigations, we aim to clarify whether TLR4 is a key upstream target of SSA’s antidepressant actions, thereby extending the current understanding of its mechanisms.

In this study, we used chronic unpredictable mild stress (CUMS) to construct a depression model and evaluated whether SSA exhibits antidepressant effect through behavioral experiments. Behavioral assessments were conducted to evaluate the efficacy of SSA, and mechanistic studies were performed to determine the involvement of TLR4 signaling in its antidepressant effects. We further validated the role of the TLR4/NF-κB/BDNF axis in mediating SSA’s actions using both *in vivo* and *in vitro* models. This research not only uncovers a previously unreported molecular mechanism of SSA but also provides novel evidence supporting the TLR4 axis as a therapeutic target for natural antidepressants. This study aimed to gain a comprehensive insight into the antidepressant mechanism of SSA and explore its potential as a natural medicine for improving mental health.

## Materials and methods

### Chemicals

Saikosaponin A (SSA) (CAS: 20736-09-8) was obtained from MedChemExpress (New Jersey, United States) and dissolved in deionized water. Corticosterone (CORT) was obtained from Wellman Pharmaceutical Group Co., Ltd. (Anhui, China). Other chemicals were of commercial origin.

### Cell culture

PC12 cells (# CRL-1721) were purchased from American Type Culture Collection. PC12 cells were seeded in 75 cm^2^ cell-culture flasks and cultured in an incubator at 37°C under 5% CO_2_ and sub-cultured at a ratio of 1:4 when the cell density reached 10^6^. The cell experiment was divided into three groups: control group, CORT treatment group (50 μM), and SSA treatment group (CORT: 50 μM + SSA 25 μg/mL).

### Overexpressing and silencing of TLR4

TLR4 overexpression plasmids (pc-TLR4), pcDNA3.1, Si-NC, and Si-TLR4 were obtained from GeneCopoeia (Rockville, MD, United States). The reagent used for transfection was Lipofectamine 3000 (Thermo Fisher Scientific).

### Animals

C57BL/6J mice (male, weight 20–23 g) were purchased from GemPharmatech Co., Ltd. and were raised in a standard animal room (12 h light/12 h dark cycle, 22°C–24°C, relative humidity of 50%–60%). All procedures were performed according to the National Institutes of Health Guide for Care and Use of Laboratory Animals and the Northwest A&F University (N81803231). After a 2-week acclimation period, they were randomly divided into three groups. Group 1: control group, group 2: CUMS group, and group 3: SSA protective group: CUMS + SSA (20 mg/kg). Specifically, the CUMS procedure involved nine randomly applied stressors for 6 weeks, including food and water deprivation, cage tilting, constant light exposure, swimming in cold water, wet cage conditions, and restraint. These stressors were designed to induce chronic stress in the subjects (Bi et al., 2019; Liu et al., 2024), and the SSA dosage (20 mg/kg) was determined based on the literature (Feng et al., 2020; Wang et al., 2018; Wei et al., 2016). During the 6-week CUMS experiment, SSA was administered daily *via* oral gavage for the entire 6-week period.

### Behavioral tests

#### Tail suspension test

The mouse was suspended in the box below the pyroelectric infrared probe, and adhesive tape was placed at 1.5 cm from the tail tip. After the mice adapted for 1 min, the formal test was conducted. The system recorded the mouse’s immobility time within 6 min and calculated the struggling time. Struggling time was automatically calculated by the system. This test was performed under consistent light conditions (100 lux).

#### Open field test

Open field test is used to measure spontaneous movement, and the experiment is conducted in a 50 cm × 50 cm × 50 cm box. The mouse was placed in the center of box, and a camera was used to record the activity trajectory within 5 min. The arena was illuminated at an intensity of 150 lux. The distance in the central area and time in the central area were recorded and analyzed by SuperMaze software. Three trials were conducted per day for each mouse, with a minimum interval of 24 h between trials.

#### Elevated plus maze

Elevated plus maze was used to assess the anxiety state of mice. The mouse was placed in the central area (5 cm × 5 cm), the trajectory of the mouse within 5 min was recorded by SuperMaze software, and relevant indicators were calculated.

#### Marble burying test

Bedding was spread with 5 cm thickness on the bottom of a 40 cm × 30 cm × 22 cm dry and clean box, 20 glass beads were placed, and the number of glass beads buried within 30 min were recorded (more than 2/3 of the glass beads covered are considered buried).

#### H&E staining

Mouse brain tissue was immersed in 4% paraformaldehyde, and then embedded in paraffin, cut into 4-μm thick sections, dehydrated, stained with hematoxylin–eosin solution (Solarbio, #G1120), and observed under a fluorescence microscope (Nikon, Japan, TE2000-E).

#### Immunohistochemistry

Paraffin-embedded brain sections were dewaxed, rehydrated, and subjected to antigen retrieval in citrate buffer (pH 6.0) *via* microwave heating (10 min). Endogenous peroxidase activity was blocked with 3% H_2_O_2_ (10 min), followed by 5% BSA blocking (30 min, RT). Sections were incubated overnight at 4°C with anti-TLR4 antibody (Abcam, ab22048, 5 μg/mL) and then with a biotinylated secondary antibody, and visualized using a DAB kit (Abcam, ab64238) (Zhao et al., 2020). Hematoxylin was used for counterstaining. Imaging was performed on a Nikon TE2000-E fluorescence microscope.

#### Western blot

Protein was extracted from mouse hippocampus using a RIPA lysis buffer (Beyotime, #P0013B) containing protease and phosphatase inhibitors. The protein concentration was determined using a BCA Assay Kit (Beyotime, #P0010S), and Western blotting experiments were performed according to the previous procedures (Zhang et al., 2021). The primary antibodies used were as follows: TLR4 (Invitrogen, 76B357.1), NF-κB (ab32536, 1/5000), BDNF (ab108319, 1/5000), and β-Actin (Abcam, ab8227, 1/5000). Protein bands were visualized using an ECL kit (Beyotime, #P0018S) and quantified using ImageJ software.

#### ELISA

The levels of indicators of inflammation and oxidative stress were measured by commercial kits (Algefare et al., 2024). ROS, SOD, GPx, GSH, GSSH, and CAT kits were all obtained from Navand Salamat Company (Urmia, Iran). MDA kits were purchased from Teb Pazhouhan Razi Company (Tehran, Iran). TNF-α, IL-1β, and IL-6 kits were obtained from Krishgen Biosystem Company (Shanghai, China).

#### Statistical analysis

SPSS 22.0 software was used for data analysis using one-way ANOVA to compare means among multiple groups, and data were expressed as mean ± standard deviation (SD). *P* < 0.05 was regarded as significant difference.

## Results

### SSA treatment inhibited CUMS-caused depressive-like behavior in mice

**FIGURE 1:**
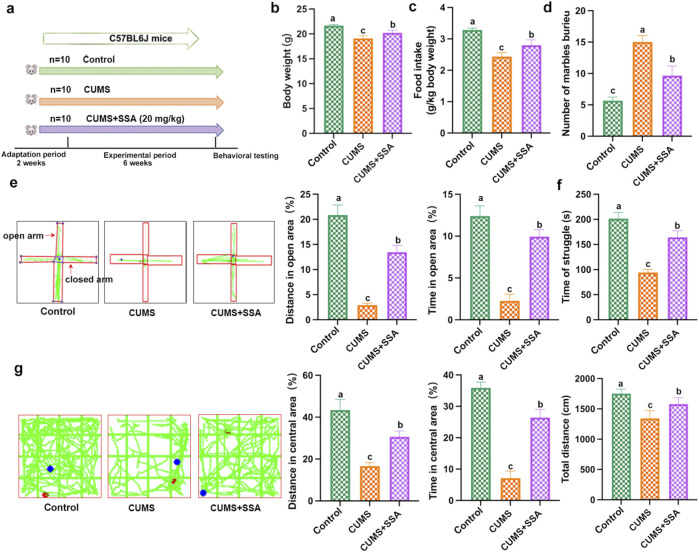
SSA protects against CUMS-induced depression in mice. **(a)** Experimental design, **(b)** body weight, **(c)** food intake, **(d)** marble burying test, **(e)** elevated plus maze test, **(f)** tail suspension test, and **(g)** open field test. Different letters indicated significant differences (*P* < 0.05).

As shown in Figure 1a, we explored the effect of SSA on CUMS-caused depressive-like behavior in mice. Figures 1b,c show that CUMS induced a decrease in mice body weight and food intake, whereas SSA treatment inhibited these changes. The marble burying test showed that SSA reduced the number of marbles buried in depressed mice (Figure 1d). Elevated plus maze showed that SSA treatment upregulated the distance in open area and time in open area in depressed mice (Figure 1e). Tail suspension test (Figure 1f) and open field test (Figure 1g) demonstrated that SSA treatment increased the time of struggle, the total distance, the distance in central area, and the time in central area compared with CUMS-treated mice.

### SSA treatment alleviated CUMS-caused hippocampus histopathological damage

**FIGURE 2:**
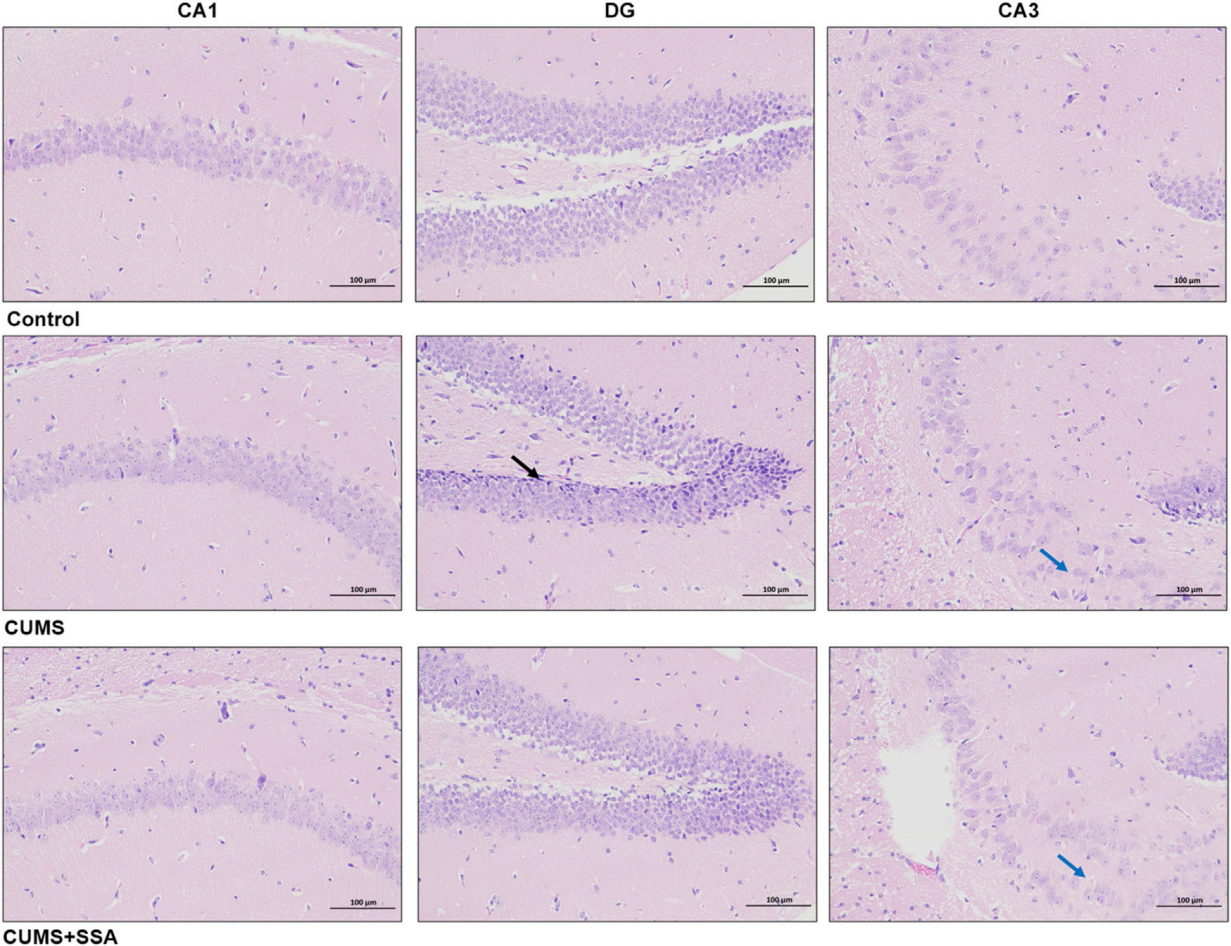
SSA mitigates CUMS-caused histopathological injury in the mouse hippocampus. H&E staining (200×); black arrows indicate shrunken granule cells with deepened staining and unclear nuclear/cytoplasmic boundaries; blue arrows show loosely arranged pyramidal cells in the CA3 region.

As shown in Figure 2, no pathological damage phenomenon was observed in the hippocampus of normal mice. In the hippocampus of CUMS-caused depressed mice, we observed that the granule cells were shrunk in the DG area (black arrows), cell staining was deepened, and the nuclear and cytoplasmic boundaries were unclear; the pyramidal cells in the CA3 area were loosely arranged (blue arrow). Whereas SSA treatment mitigated the pathological damage in the hippocampus of CUMS-caused depressed mice, the pyramidal cells in the hippocampus have regular morphology, and only a small number of pyramidal cells were observed to be loosely arranged in the CA3 area (blue arrow).

### SSA treatment inhibited CUMS-caused disorder of neurotransmitters and TLR4/NF-κB/BDNF axis

**FIGURE 3:**
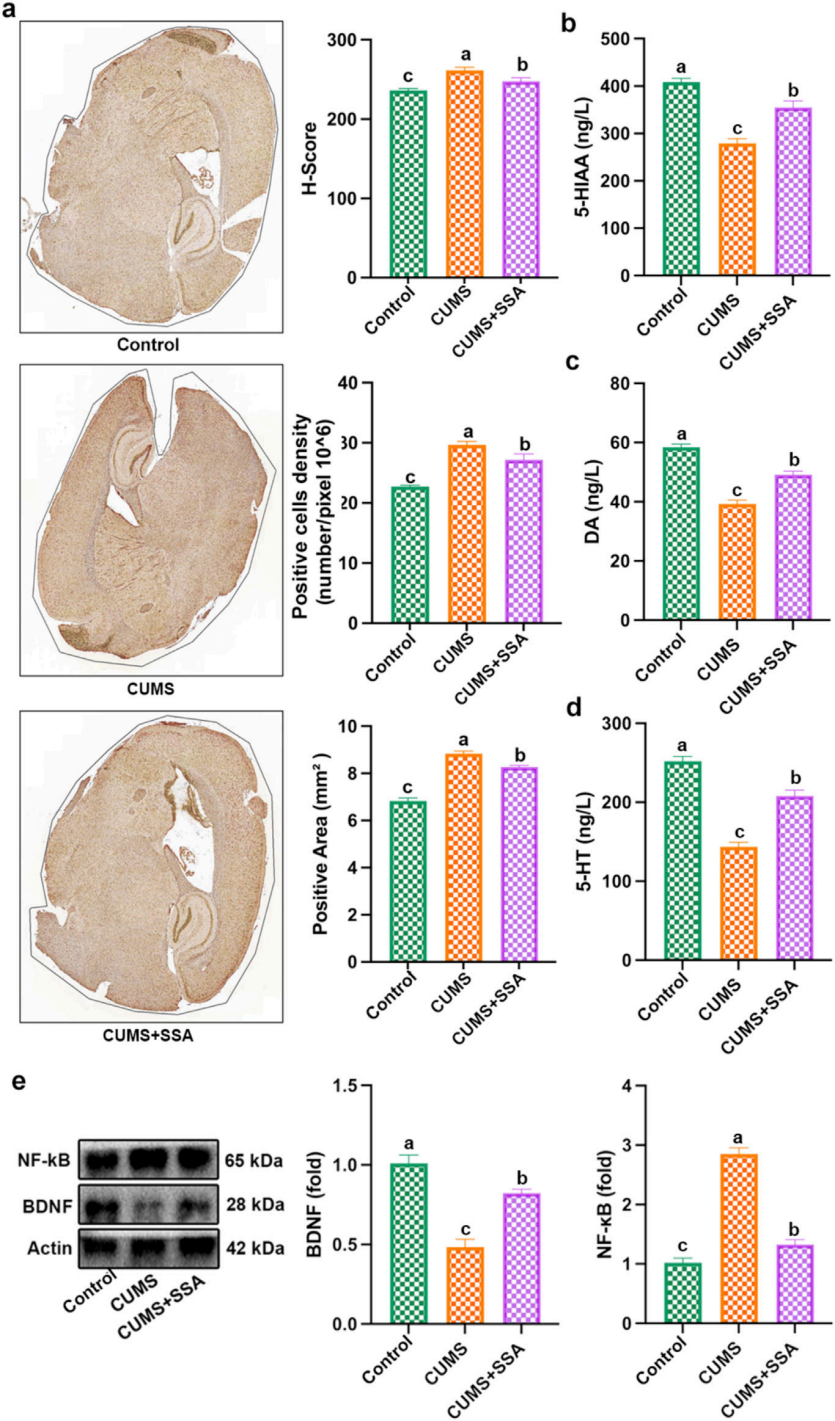
SSA treatment inhibited CUMS-caused disorder of neurotransmitters and the TLR4/NF-κB/BDNF axis. **(a)** Immunohistochemistry test of TLR4, **(b)** 5-HIAA level, **(c)** MDA level, **(d)** 5-HT level, and **(e)** protein levels of BDNF and NF-κB. Different letters indicated significant differences (*p* < 0.05).

As shown in Figure 3a, we detected the level of TLR4. The results indicated that CUMS upregulated the TLR4 level, whereas SSA treatment downregulated it. Figures 3b–d show that SSA treatment increased the levels of neurotransmitters, including 5-HT, 5-HIAA, and DA. Figure 3e indicates that SSA treatment reduced the NF-κB protein level and increased the BDNF protein level. These results demonstrated that SSA treatment mitigated CUMS-caused disorders of neurotransmitters and TLR4/NF-κB/BDNF axis.

### SSA treatment inhibited CUMS-caused oxidative damage and inflammation

**FIGURE 4:**
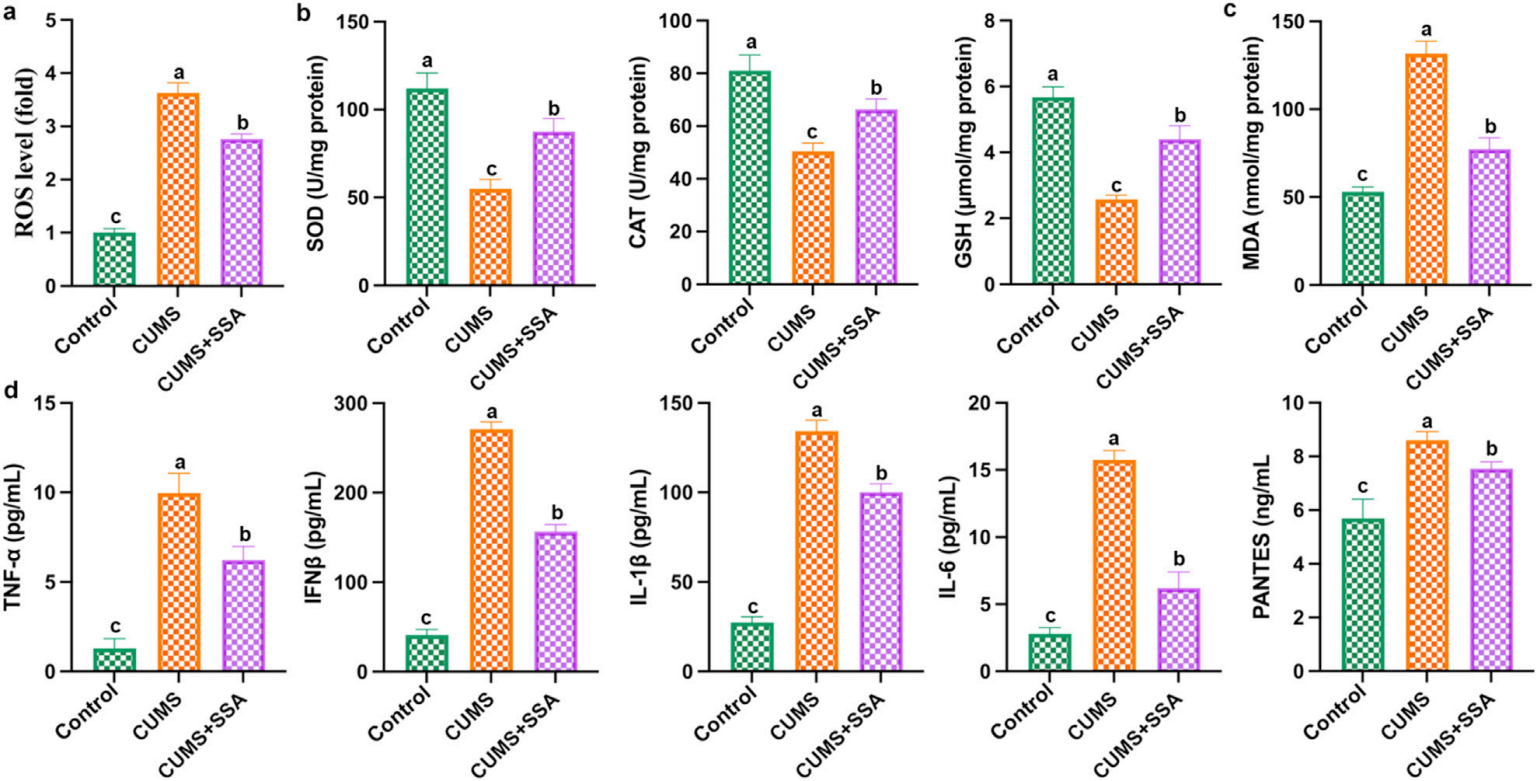
SSA alleviates CUMS-caused oxidative damage and inflammation. **(a)** ROS level; **(b)** SOD, CAT, and GSH levels; **(c)** MDA level; and **(d)** IL-6, IFNβ, IL-1β, PANTES, and TNF-α levels in brain lysates. Different letters indicated significant differences (*p* < 0.05).

As shown in Figure 4, we measured the levels of oxidative stress and inflammation-related mediators. The results show that CUMS caused an increase in the levels of ROS and MDA and the inflammatory factors, including IL-6, IFNβ, IL-1β, PANTES, and TNF-α, and caused a decrease in the levels of GSH, SOD, and CAT in mice hippocampus, whereas SSA treatment inhibited these changes, which demonstrated that SSA treatment mitigated CUMS-caused oxidative stress and inflammation.

### TLR4 overexpression attenuated the effect of SSA on CORT-induced TLR4/NF-κB/BDNF axis dysregulation

**FIGURE 5:**
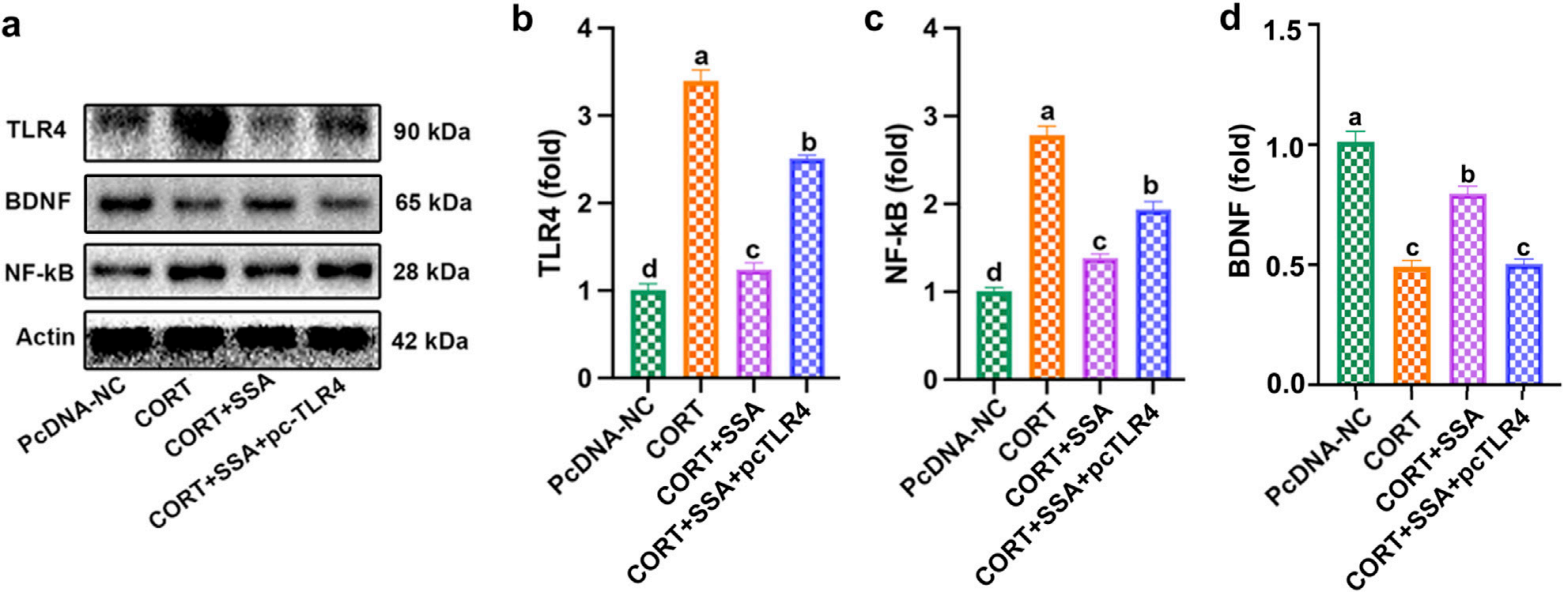
TLR4 overexpression attenuated the effect of SSA on CORT-induced TLR4/NF-κB/BDNF axis dysregulation. **(a)** Western blot test, **(b)** TLR4 protein level, **(c)** NF-κB protein level, and **(d)** BDNF protein levels. Different letters indicated significant differences (*p* < 0.05).

In this study, we constructed PC12 cell models using CORT to explore the antidepressive effect of SSA. As shown in Figure 5, SSA attenuated CORT-caused increases in TLR4 and NF-κB protein levels and decreases in BDNF protein levels, and TLR4 overexpression increased TLR4 and BDNF protein levels compared to SSA-treated group. These results indicated that TLR4 overexpression attenuated the effect of SSA on CORT-induced TLR4/NF-κB/BDNF axis dysregulation.

### TLR4 overexpression attenuated the effect of SSA on CORT-induced PC12 cell damage, oxidative stress, and inflammation

**FIGURE 6:**
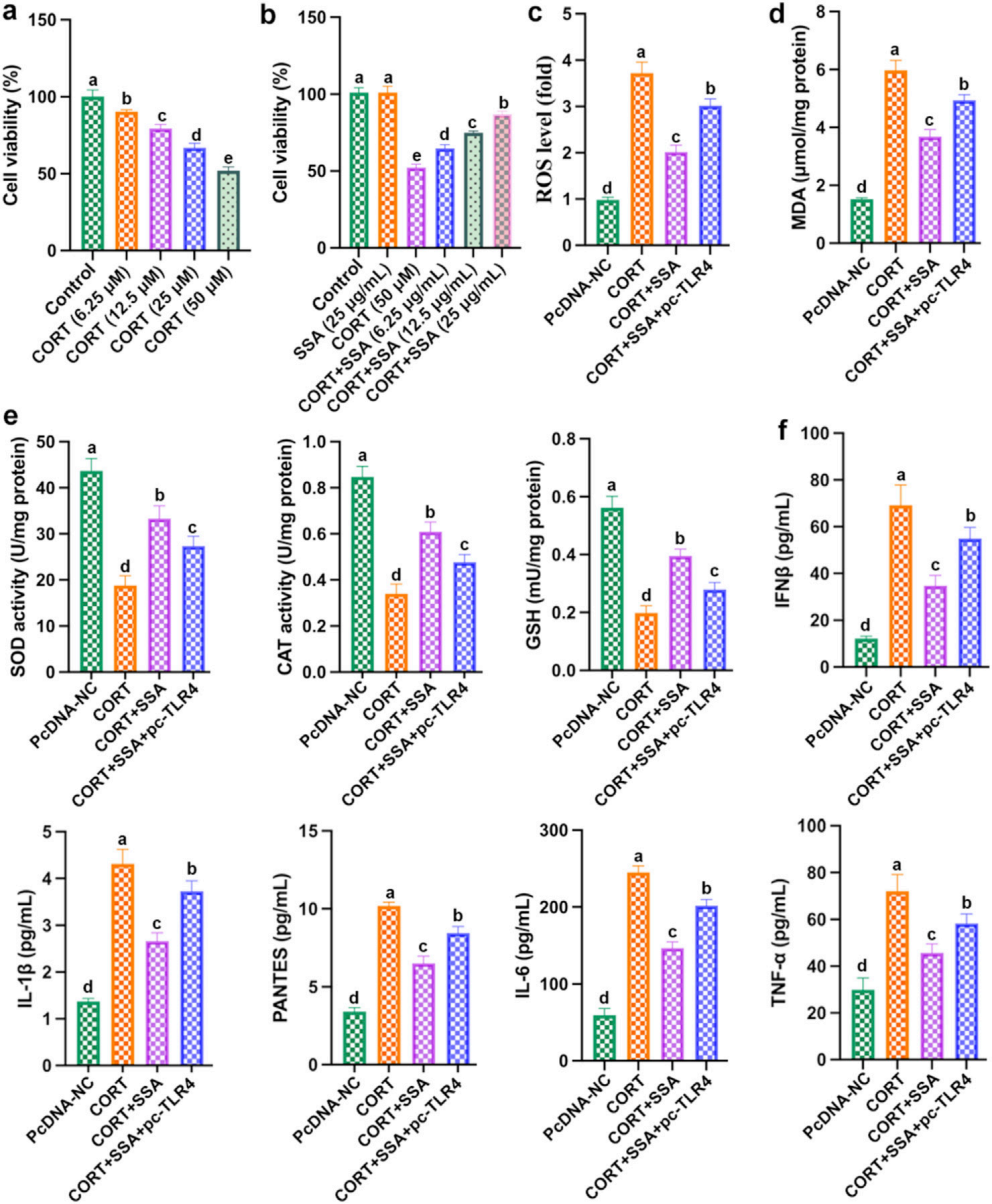
Overexpression of TLR4 weakened the protective effect of SSA. **(a)** Cell viability; **(b)** cell viability; **(c)** ROS level; **(d)** MDA content; **(e)** levels of SOD, CAT, and GSH activities; and **(f)** levels of IL-6, IFNβ, IL-1β, PANTES, and TNF-α. Different letters indicated significant differences (*p* < 0.05).

As shown in Figure 6a, as the concentration of CORT increased, PC12 cell viability gradually reduced, and the cell viability dropped to approximately 50% at the high dose of 50 μM. As shown in Figure 6b, compared with the CORT group, PC12 cells’ viability increased accordingly as the SSA concentration was upregulated, and the SSA concentration of 25 μg/mL had the best protective effect. These results demonstrated that SSA attenuates CORT-treated PC12 cells in a dose-dependent manner. Subsequently, 50 μM of CORT and 25 μg/mL of SSA were selected as the subsequent experiment. Furthermore, we transfected TLR4 overexpression plasmid (pc-TLR4) into CORT and SSA-treated PC12 cells. As shown in Figures 6c–f, the overexpression of TLR4 attenuated the protective effect of SSA, increased the levels of inflammatory factors (IL-6, IFNβ, IL-1β, PANTES, and TNF-α), and reduced the levels of antioxidant enzymes (GSH, SOD, and CAT). These findings strongly suggest that SSA alleviates CORT-caused PC12 cell damage and depression by modulating TLR4-related oxidative stress and inflammation.

### TLR4 silencing mitigated CORT-caused PC12 cell damage by inhibiting oxidative stress and inflammation

**FIGURE 7:**
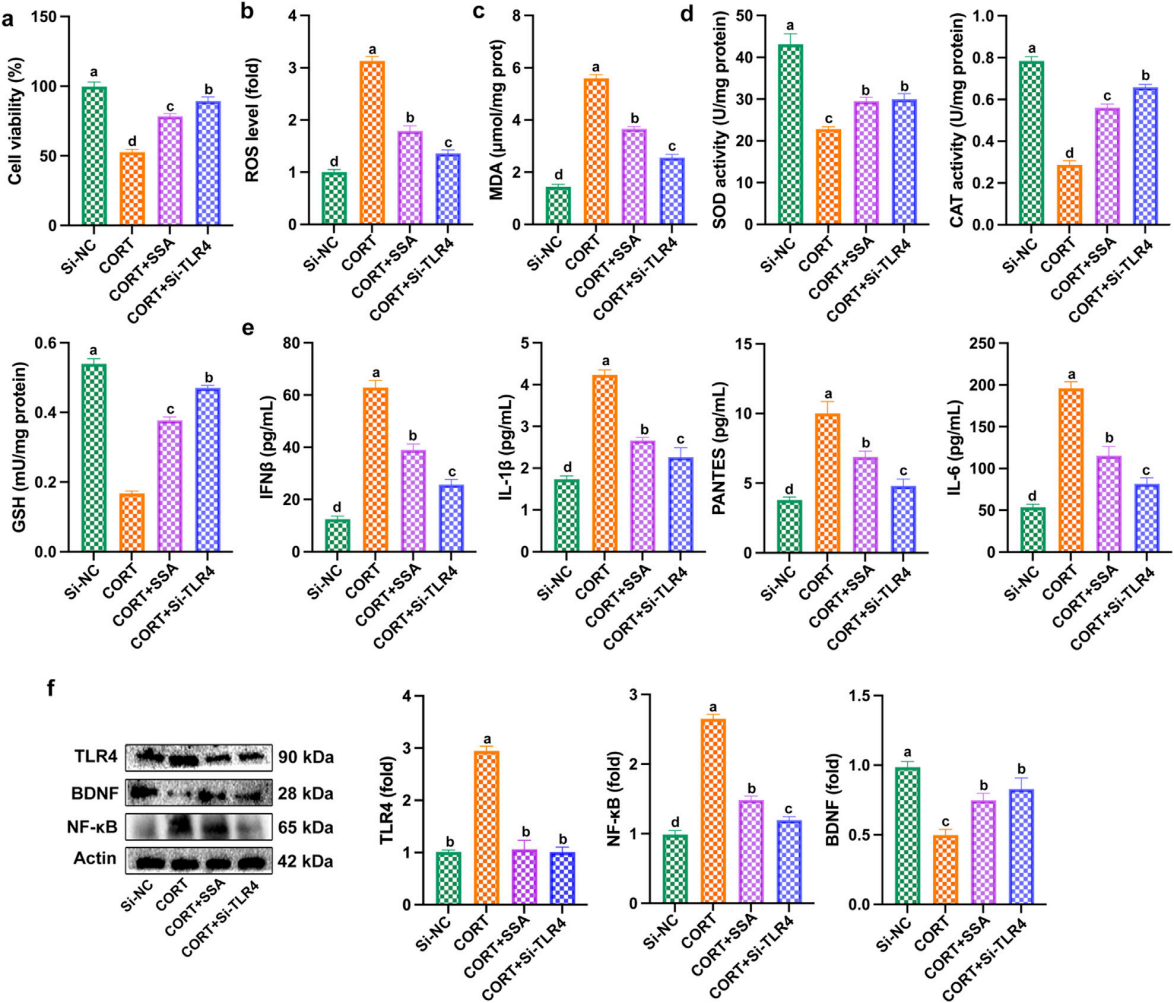
Silencing of TLR4 mitigated CORT-caused PC12 cell damage, oxidative stress, and inflammation. **(a)** Cell viability; **(b)** ROS level; **(c)** MDA content; **(d)** levels of SOD, CAT, and GSH activities; **(e)** levels of IL-6, IFNβ, IL-1β, PANTES, and TNF-α; and **(f)** Western blot test. Different letters indicated significant differences (*p* < 0.05).

As shown in Figures 7a–c, silencing TLR4 in CORT-treated PC12 cells significantly increased cell survival and reduced ROS and MDA levels. At the same time, the activities of antioxidant enzymes (SOD, CAT, and GSH) were increased, and the levels of inflammatory factors (IL-6, IFNβ, IL-1β, PANTES, and TNF-α) were reduced (Figures 7d,e). In addition, as shown in Figure 7f, silencing TLR4 downregulated the levels of TLR4 and NF-κB proteins and upregulated the levels of BDNF proteins in CORT-treated PC12 cells. The results showed that the effect of silencing TLR4 was similar to that of SSA treatment, and both were able to alleviate CORT-induced PC12 cell damage through inhibiting inflammation and oxidative stress.

## Discussion and conclusion

**FIGURE 8:**
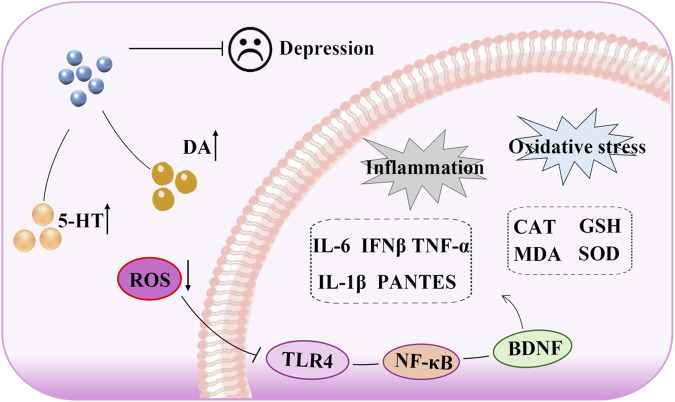
Antidepressant mechanism of saikosaponin A: the role of TLR4/NF-κB/BDNF axis-mediated oxidative stress and inflammation.

In this study, we evaluated CUMS-induced depressive-like behaviors using a battery of behavioral tests, and our results demonstrated that SSA exerted a significant antidepressant effect. Through both *in vivo* and *in vitro* analyses, we further demonstrated that SSA attenuated neuroinflammation and oxidative stress by modulating the TLR4/NF-κB/BDNF signaling pathway, identifying TLR4 as a potential therapeutic target (Figure 8).

Neurotransmitters play a critical role in regulating the behavior and physiological functions of the central nervous system (Pahlavani, 2024). Studies have found that the disorder of monoamine neurotransmitters is closely related to depression, and the occurrence of depression is often accompanied by a decrease in neurotransmitter levels (Fitzgerald, 2024). Among these, DA and 5-HT are the most important types of monoamine neurotransmitters in the brain, and their functions involve mood regulation. Chen et al. (2018) found that CUMS induced depressive-like behavior in rats, which reduced the levels of DA, HVA, 5-HT, and 5-HIAA in brain homogenates of rats (Chen et al., 2019). Consistent with these findings, our study showed that SSA significantly improved depressive-like behaviors in CUMS-exposed rats, enhanced behavioral performance, and restored the levels of 5-HT, 5-HIAA, and DA. These results suggest that SSA may exert its antidepressant effects by modulating monoamine neurotransmitter metabolism.

Previous studies have shown that the activation of oxidative stress and inflammation contributes to the development of depression, and antidepressant treatments are usually accompanied by the inhibition of neuroinflammation and oxidative stress (Bhatt et al., 2020; Moon et al., 2018). Tsai and Huang (2016) reported that oxidative damage in the brain of depressed patients correlates with decreased antioxidant levels (such as GSH and GST) and elevated MDA levels (Tsai and Huang, 2016). Furthermore, depression is often accompanied by neuroinflammation, which is characterized by elevated pro-inflammatory cytokines. A recent meta-analysis revealed that depression was associated with the levels of inflammatory factors (such as IFN-α2, IFN-γ, IL-12, IL-19, IL-35, IL-32, IL-2, IL-11, and IFN-β) and oxidative stress (such as SOD, CAT, T-AOC, and GPx) (Wen et al., 2024). In this study, CUMS caused abnormal increase in ROS and MDA levels in the hippocampus of mice, decreased levels of antioxidant enzymes (including SOD, CAT, and GSH), and increased levels of inflammatory factors (TNF-α, IFNB, PENTES, IL-1β, and IL-6), whereas SSA significantly inhibited these changes, indicating that SSA mitigated the occurrence of depression-induced oxidative stress and neuroinflammation.

BDNF, as an important neurotrophic factor, is involved in the occurrence of neurological diseases such as depression (Yang T. et al., 2020) and exerted the ability to regulate neuroinflammation and oxidative stress (Birmann et al., 2022). The increase in the number of BDNF-positive cells in the hippocampus and cerebral cortex can slow down the occurrence of inflammatory responses in the brains of aged mice (Elahi et al., 2016). Conversely, elevated ROS levels impair neuronal integrity, promote cellular damage, and suppress BDNF expression (Murugan et al., 2024). ROS not only triggers oxidative stress (Briyal et al., 2023) but also promotes inflammation, thereby exacerbating depressive symptoms (Olugbemide et al., 2021; Shen et al., 2021; Zhao et al., 2021). In our study, SSA significantly reduced ROS levels and increased BDNF expression, suggesting that SSA may alleviate depression by reducing oxidative stress and neuroinflammation, at least in part through upregulating BDNF.

TLR4, a member of the TLR family, plays a crucial role in mediating inflammatory responses by triggering the production of various pro-inflammatory mediators (Zhang et al., 2022). Dysregulation of the TLR4 signaling pathway has been linked to numerous diseases, including sepsis and cardiovascular conditions (Bezhaeva et al., 2022; Li et al., 2023). It is worth noting that the TLR4 pathway plays a critical role in regulating depression-related inflammation and oxidative stress (Abdelzaher et al., 2021). Puerarin inhibits inflammation and phospholipid metabolism disorder by regulating the TLR4-mediated cPLA2/COX-2 axis, thereby alleviating the depressive-like behavior caused by high-fat diet and CUMS (Gao et al., 2021). Liquiritin could attenuate the occurrence of CUMS-induced oxidative stress and inflammation through its antioxidant and anti-inflammatory properties, thereby intervening in depressive-like behavior (Liu et al., 2022). In this study, we found that SSA treatment inhibited depression-caused increases in the TLR4 level and that the TLR4/NF-κB axis was involved. Additionally, we overexpressed TLR4 in PC12 cells, and the results showed that overexpression of TLR4 weakened the antidepressant effect of SSA, which enhanced inflammation, oxidative stress, and depressive-like behavior. Furthermore, silencing TLR4 reduced CORT-caused PC12 cell damage, which was evidenced by the cell viability and indicators of inflammation and oxidative stress. In conclusion, the results demonstrated that SSA could inhibit CORT-caused depression through regulating TLR4/NF-κB/BDNF axis-mediated inflammation and oxidative stress. This study clarified the antidepressant mechanism of SSA and discovered that TLR4 can be used as a key target for antidepression therapies, providing new directions and ideas for the future prevention and treatment of depression and the development of target drugs.

## Data Availability

The original contributions presented in the study are included in the article/supplementary material; further inquiries can be directed to the corresponding author.
